# Obeticholic acid and ferrostatin-1 differentially ameliorate non-alcoholic steatohepatitis in AMLN diet-fed ob/ob mice

**DOI:** 10.3389/fphar.2022.1081553

**Published:** 2022-12-16

**Authors:** Shengjie Li, Aoxiang Zhuge, Kaicen Wang, Jiafeng Xia, Qiangqiang Wang, Shengyi Han, Jian Shen, Lanjuan Li

**Affiliations:** ^1^ State Key Laboratory for Diagnosis and Treatment of Infectious Diseases, National Clinical Research Centre for Infectious Diseases, Collaborative Innovation Centre for Diagnosis and Treatment of Infectious Diseases, The First Affiliated Hospital, Zhejiang University School of Medicine, Hangzhou, China; ^2^ Jinan Microecological Biomedicine Shandong Laboratory, Jinan, China

**Keywords:** obeticholic acid, ferrostatin-1, steatohepatitis, oxidative stress, fibrosis, microbiome, lipidomics

## Abstract

**Introduction:** Non-alcoholic fatty liver disease (NAFLD) and non-alcoholic steatohepatitis (NASH) are common chronic liver diseases with limited treatment options.

**Methods:** Ob/ob mice (6 weeks old) were fed with the Control diet or amylin liver NASH (AMLN) diet for 24 weeks to establish the NASH, the AMLN diet-fed mice were treated with obeticholic acid (OCA), ferrostatin-1 (Fer-1) or their combination for 7 weeks. Finally, various clinical profiles were assessed.

**Results:** Our results indicate that Fer-1 exerts better effects on improving body weight, blood glucose levels, transaminase levels and insulin resistance than OCA. OCA has a profound effect on ameliorating lipid accumulation. OCA and Fer-1 differentially inhibit the activation of hepatic Kupffer cells and HSCs. The combination of OCA and Fer-1 significantly reduces inflammation and protects mice against liver oxidative stress. OCA and Fer-1 differentially reshape the intestinal microbiota and affect the hepatic lipidome.

**Discussion:** Our study compares the effects of OCA, Fer-1 and their combination on various clinical profiles in NASH. These data demonstrate that different drug combinations results in different improvements, and these discoveries provide a reference for the use of the OCA, Fer-1 and their combination in the clinical treatment of NAFLD/NASH.

## 1 Introduction

Non-alcoholic fatty liver disease (NAFLD), which is featured with hepatic steatosis and fibrosis, is a common chronic liver disease worldwide (Friedman et al., 2018). The prevalence of NAFLD is growing with the growth of obesity and diabetes, and the global prevalence is currently estimated to be 25% (Younossi et al., 2018; Younossi, 2019). As the disease progresses, NAFLD may develop into non-alcoholic steatohepatitis (NASH), which is characterized by inflammation and fibrosis; worse, these liver injuries may lead to cirrhosis, liver failure and liver cancer (Margini and Dufour, 2016). Due to the high risk of morbidity and the substantial burden on the healthcare system, it is urgent to search for more effective treatments.

Researchers have found that multiple factors participate in the pathogenesis and progression of NASH (Friedman et al., 2018). Currently, there is still a lack of drugs that are approved by the Food and Drug Administration (FDA) to treat NAFLD/NASH, but some pharmacological agents, such as insulin sensitizers, antioxidants, lipotoxicity-based targets, gastrointestinal hormones and molecules that modulate nuclear transcription factors, have been considered to be alternative therapeutics (Raza et al., 2021). Obeticholic acid (OCA), an effective Farnesoid X receptor (FXR) agonist which is derived from chenodeoxycholic acid, improves NAFLD/NASH by regulating bile acid synthesis and enterohepatic circulation in the body (Mudaliar et al., 2013). A multicenter, double-blind, placebo-controlled clinical trial of NASH patients without cirrhosis indicated that OCA increased glucose levels and insulin resistance (Neuschwander-Tetri et al., 2015). The phase 3 trial showed that OCA (25 mg) significantly improved fibrosis and key components of NASH among patients, but it also triggered an adverse event (pruritus) (Younossi et al., 2019). It was reported that OCA administration increased the risk of gallstone formation (Al-Dury et al., 2019). Thus, OCA ameliorates NASH in diverse ways and shows great potential, but the side effects also attracted our attention.

It is generally accepted that hepatocytes that are damaged by lipotoxicity release proinflammatory factors and produce hepatotoxic substances that induce stress responses, leading to NASH (Diehl and Day, 2017). Ferroptosis, which is induced by the iron-dependent accumulation of lipid reactive oxygen species (ROS), is a recently discovered form of cell death (Dixon et al., 2012) and was recently found to be correlated with the onset of NASH (TsurUnited stateski et al., 2019). In support of this view, ferroptosis inhibitors, such as liproxstatin-1, were proven to alleviate hepatic lipid peroxidation and the related cell death in methionine/choline-deficient diet (MCD)-treated mice (Qi et al., 2020). Moreover, ferrostatin-1 (Fer-1) was reported to ameliorate MCD diet-induced inflammation, fibrosis and liver injury in mice with NASH (Li et al., 2020). However, the impact of Fer-1 on NAFLD/NASH, as well as the underlying mechanism, still require further research.

A variety of drugs may influence different metabolic and molecular pathways (Fan et al., 2021; Raza et al., 2021; Lin et al., 2022); for this reason, a combination of treatments could be beneficial to patients by targeting diverse pathological profiles. We hypothesize that compared to OCA, Fer-1 could additively improve and affect the progression and prognosis of NASH through different mechanisms. Our study investigates and compares how OCA and Fer-1, alone or in combination, affects liver histology, lipid accumulation, and hepatic Kupffer and stellate cell (HSC) activation as well as the impacts of these drugs on the gut microbiota and liver lipidomics in a mouse model of NASH induced by amylin liver NASH (AMLN) diet.

## 2 Materials and methods

### 2.1 Experimental design

**FIGURE 1:**
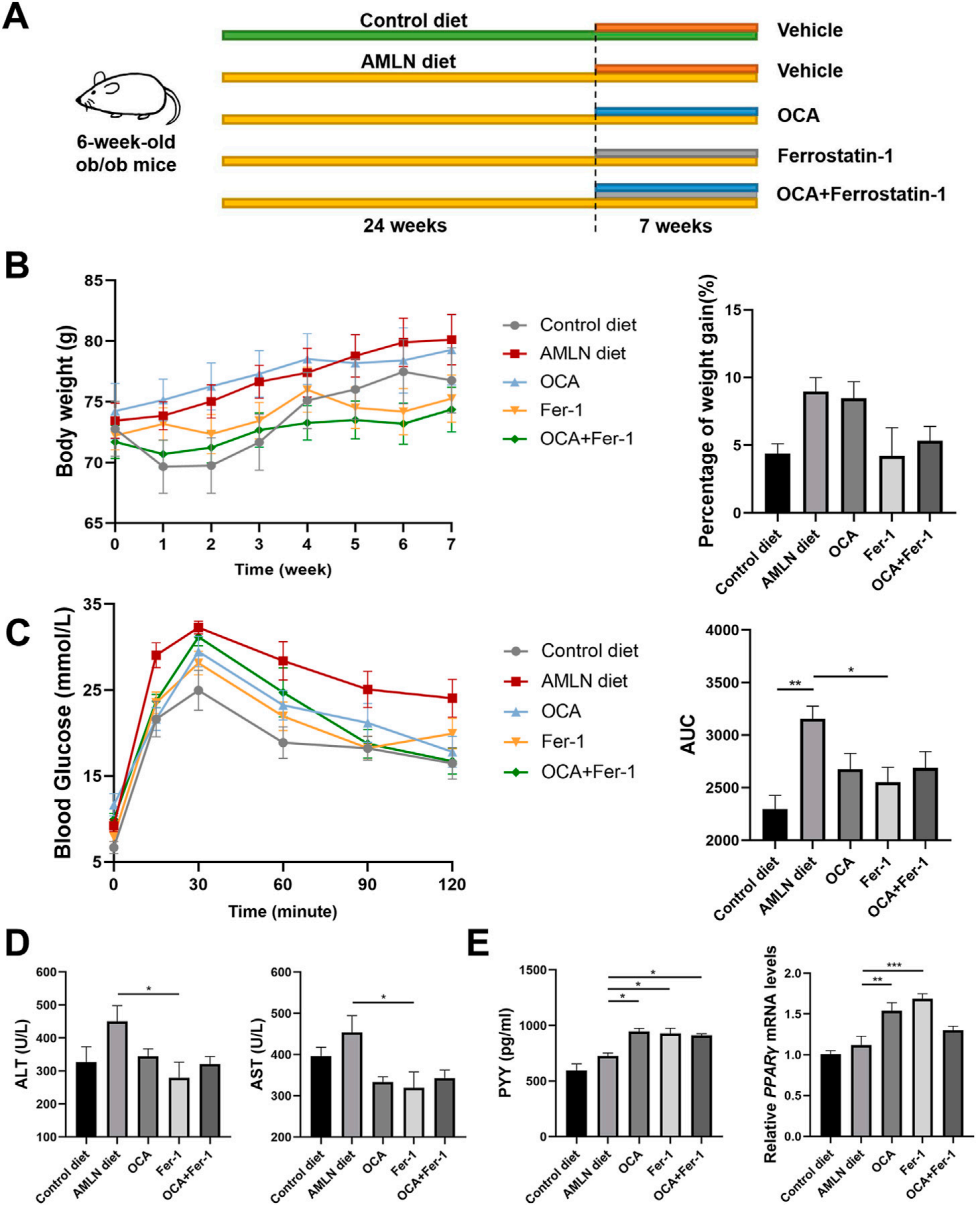
Fer-1 exhibited better effects on improving metabolic status than OCA in AMLN diet induced NASH mice. **(A)** Experimental scheme. **(B)** Body weight after treatments (left) and the percentage of weight gain after treatments (right). **(C)** Blood glucose (left) and area under the curve (right) of IGTT. **(D)** Liver function tests of serum (ALT and AST). **(E)** Levels of PYY in the liver (left) and relative expression of *PPARγ* in liver tissues. Data are shown as mean ± SEM. *p < 0.05, **p < 0.01, ***p < 0.001.

Ob/ob mice (6 weeks old, *n* = 40) were obtained from GemPharmatech (Nanjing, China) and kept in a specific pathogen-free environment with free access to water and food. After 1 week of adaptation, the mice were randomly divided into two groups. The mice in the first group were given a control diet for 24 weeks (Control diet group, *n* = 9), while the mice in the other group were given an AMLN diet (Fanbo Biotechnology, Shanghai, China), which consisted of 40% kcal fat, 20% kcal fructose and 2% w/w cholesterol. In week 24, the mice that were fed the AMLN diet were randomly regrouped into four new subgroups: the AMLN diet group (*n* = 9), OCA group (*n* = 9), Fer-1 group (*n* = 9) and OCA + Fer-1 group (*n* = 10). Then, the mice in the OCA group and OCA + Fer-1 group were gavaged with obeticholic acid (OCA, sunshine Chemical, Wuhan, China) at a dose of 30 mg/kg (dissolved in 0.1% methylcellulose solution) daily for 7 weeks, while the mice in the other three groups were gavaged with the same volume of vehicle. Moreover, the mice in the Fer-1 group and OCA + Fer-1 group were intraperitoneally injected with ferrostatin-1 (Fer-1, Selleck, Houston, Texas, United States) at a dose of 1 mg/kg (dissolved in 0.1% DMSO solution) daily for 7 weeks, while the mice in the other three groups were intraperitoneally injected with the same volume of vehicle. A brief schedule of the mice experiment is displayed in Figure 1A. At the end of week 31, all the mice were sacrificed after anesthesia (mice were intraperitoneally injected with sodium pentobarbital at a dose of 50 mg/kg, and then sacrificed after being confirmed to remain supine and the eyelid reflexes disappeared). The blood, liver tissue and cecal contents were collected then stored at −80 C until further use. All the experiments were approved by the Animal Care and Use Committee of the First Affiliated Hospital, School of Medicine, Zhejiang University (Permit number: 2021-1411).

### 2.2 Glucose tolerance and insulin resistance analysis

An intraperitoneal glucose tolerance test (IGTT) was conducted in week 30. Mice were fasted overnight (16 h), then fasting blood glucose levels (0 min) were measured the next morning using a glucometer (Roche, Basel, Switzerland). Then, the mice were intraperitoneally administered 2 g/kg glucose, after which blood glucose levels at different time points were measured (15 min, 30 min, 60 min, and 90 min). The levels of peptide tyrosine tyrosine (PYY) and glucagon-like peptide-1 (GLP-1) in the liver were assessed by enzyme-linked immunosorbent assay (ELISA) (RayBiotech, Atlanta, United States).

### 2.3 Serum parameter analysis

Blood was centrifuged at 3000 rpm for 15 min at 4 C, then, serum was collected for serum parameter analysis. The serum levels of alanine aminotransferase (ALT), aspartate aminotransferase (AST), total cholesterol (TC), triglyceride (TG) and total bile acid (TBA) were measured by an automatic biochemistry analyzer (Mindray, BS-220, Shenzhen, China). A Mouse Bio-Plex Pro Assay (23-Plex Panel, Bio-Rad, California, United States) was conducted to measure the serum levels of IL-12 (p40), IL-13, IL-17, TNF-α, keratinocyte chemoattractant (KC) and monocyte chemoattractant protein-1 (MCP-1) following the manufacturer’s protocol.

### 2.4 Histological examination and immunohistochemical staining

Liver tissues were fixed in 10% formalin (24 h), embedded in paraffin, cut into sections (2 μm), subjected to hematoxylin and eosin (H&E) staining or Sirius red staining, and imaged under a microscope. Immunohistochemical staining for α-smooth muscle actin (α-SMA) was conducted using an anti-α-SMA antibody (Abcam, Cambridge, United Kingdom, 1/400 dilution) following the standard procedure. ImageJ (Rawak Software, Stuttgart, Germany) was used to analyze the percentage of positive area.

### 2.5 Dihydroethidium (DHE) staining and oil red O staining

Liver tissues were fixed in 4% paraformaldehyde (24 h), placed in 15% sucrose solution at 4 C and then transferred to 30% sucrose solution at 4 C for dehydration and sedimentation. The dehydrated tissues were embedded in OCT solution, placed on a quick-freezing table, cut into sections (8–10 μm), subjected to DHE and DAPI staining, and then observed under a fluorescence microscope. Oil red O staining was conducted with oil red O dye and hematoxylin dye using frozen sections. ImageJ (Rawak Software, Stuttgart, Germany) was used to analyze the average fluorescence intensity.

### 2.6 Estimation of hepatic oxidative stress

Liver levels of superoxide dismutase (SOD), malondialdehyde (MDA) and glutathione (GSH) were measured by ELISA (Sigma‒Aldrich, St. Louis, MO, United States; Abcam, Cambridge, United Kingdom; Beyotime, Shanghai, China).

### 2.7 Real-time PCR analysis

A RNeasy Plus Kit (Qiagen, Dusseldorf, Germany) was applied to conduct RNA extraction of the liver tissues according to the manufacturer’s recommendations. Subsequently, the extracted RNA was converted to cDNA using a reverse transcription assay (Takara, Tokyo, Japan) under the required conditions. A ViiA7 real-time PCR system (Applied Biosystems) was used to determine the relative expressions of target genes (normalized to the housekeeping gene GAPDH) using a SYBR reagent (Takara, Tokyo, Japan). Primer sequences used in this study are listed in the Supplementary Table S1.

### 2.8 Statistical analysis

We apply GraphPad Prism v.8 (GraphPad Software Inc, San Diego, United States) to analyze the statistical difference and draw figures. Kolmogorov-Smirnov test was used to conduct normality test. For two groups with normal distribution, student’s t-test was used to examine differences, otherwise, a Mann–Whitney U test was used; ANOVA was performed to examine more than two groups with equal SDs, otherwise, Brown-Forsythe and Welch ANOVAs were performed. The results are presented as the mean ± SEM, and difference with *p*-value <0.05 was recognized as statistically significant.

## 3 Results

### 3.1 Fer-1 exhibits better effects on improving body weight, blood glucose levels, transaminase levels and insulin resistance than OCA

In the process of AMLN-feeding, several mice were died due to NASH progression or other reasons (Supplementary Figure S1). AMLN diet-fed ob/ob mice shows steady weight gain without any treatment. OCA has little effect on weight control, while Fer-1 slows weight gain (Figure 1B). Compared to mice that were fed the control diet, AMLN diet-fed mice has obviously weaker glucose tolerance, which is characterized by higher blood glucose levels and areas under the curve (AUCs), while Fer-1 but not OCA treatment reverses this trend (Figure 1C). The same trend is observed in the serum levels of ALT and AST (Figure 1D). Insulin resistance analysis reveals that both OCA and Fer-1 significantly increase the serum level of PYY and the relative expression of PPARγ in the liver (Figure 1E). However, the serum level of GLP-1 shows no obvious changes after these treatments (Supplementary Figure S2).

### 3.2 OCA and Fer-1 significantly improve lipid accumulation and inflammation

**FIGURE 2:**
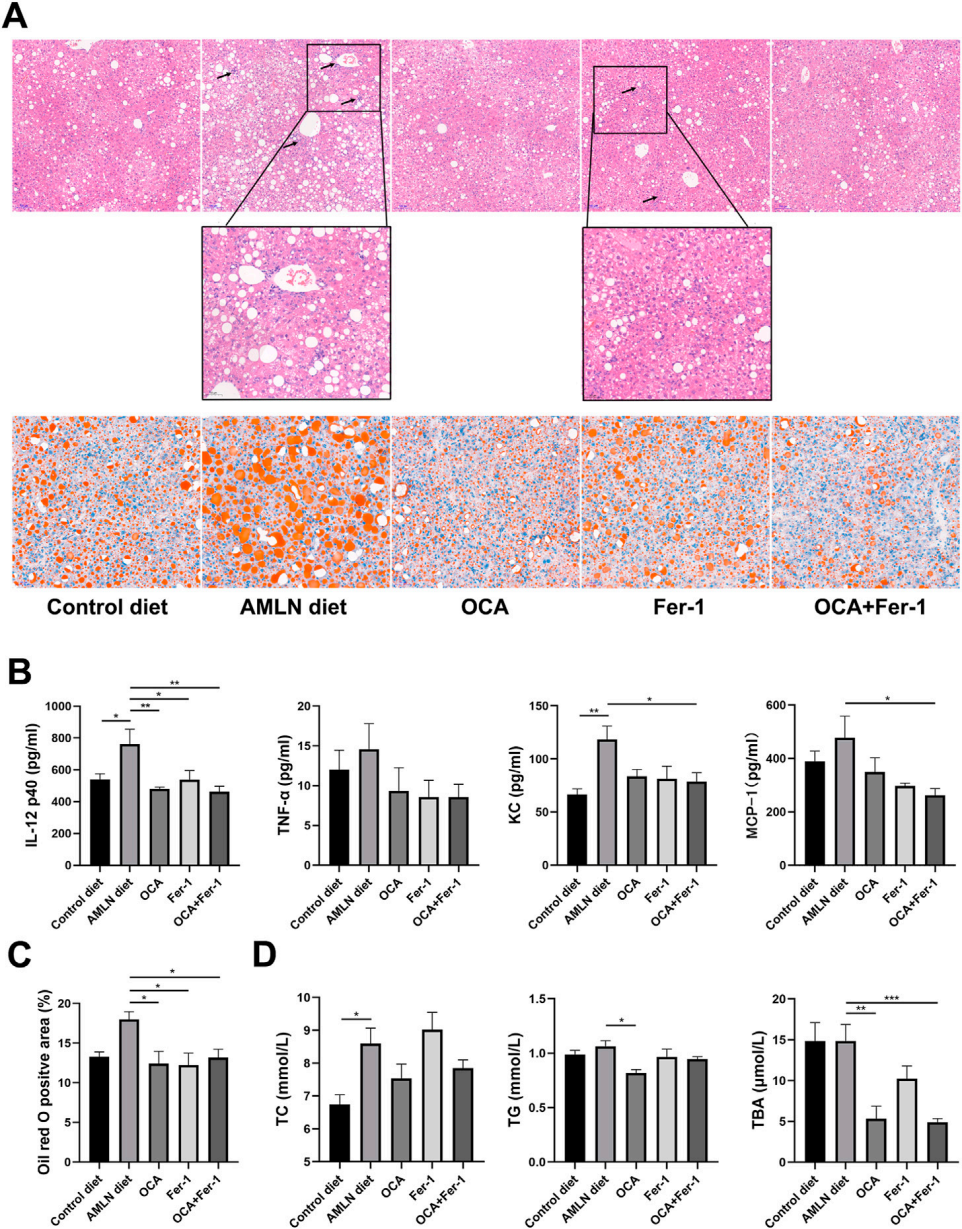
Combination of OCA and Fer-1 significantly reduced lipid accumulation and inflammation. **(A)** Representative H&E (upper, scale bar: 100 μm) and Oil red O (lower, upper, scale bar: 50 μm) staining of liver tissues, the neutrophil infiltration area was enlarged and pointed out with black arrows (scale bar: 50 μm). **(B)** Serum cytokine levels of IL-12 p40, TNF-α, KC and MCP-1. **(C)** The positive area of Oil red O staining. **(D)** Serum levels of TC, TG and TBA. Data are shown as mean ± SEM. *p < 0.05, **p < 0.01, ***p < 0.001.

As Figure 2A shows, the AMLN diet induces more severe inflammatory cell infiltration and lipid accumulation in the liver than the Control diet. H&E staining reveals that Fer-1 slightly reduces hepatic inflammation, while OCA and OCA + Fer-1 markedly alleviate hepatic inflammation. Serum analysis shows that OCA and Fer-1 differentially lower the levels of cytokines and chemokines, and cotreatment with OCA and Fer-1 significantly decreases the levels of IL-12 p40, KC and MCP-1 (Figure 2B, Supplementary Figure S3). Oil red O staining shows that both OCA and Fer-1 markedly alleviate hepatic lipid accumulation (Figure 2A, Figure 2C). Consistently, the OCA and OCA + Fer-1 treatments have a better effect on decreasing the serum levels of TC, TG and TBA than Fer-1 treatment (Figure 2D).

### 3.3 OCA and Fer-1 differentially inhibit the activation of hepatic kupfer cells and HSCs

**FIGURE 3:**
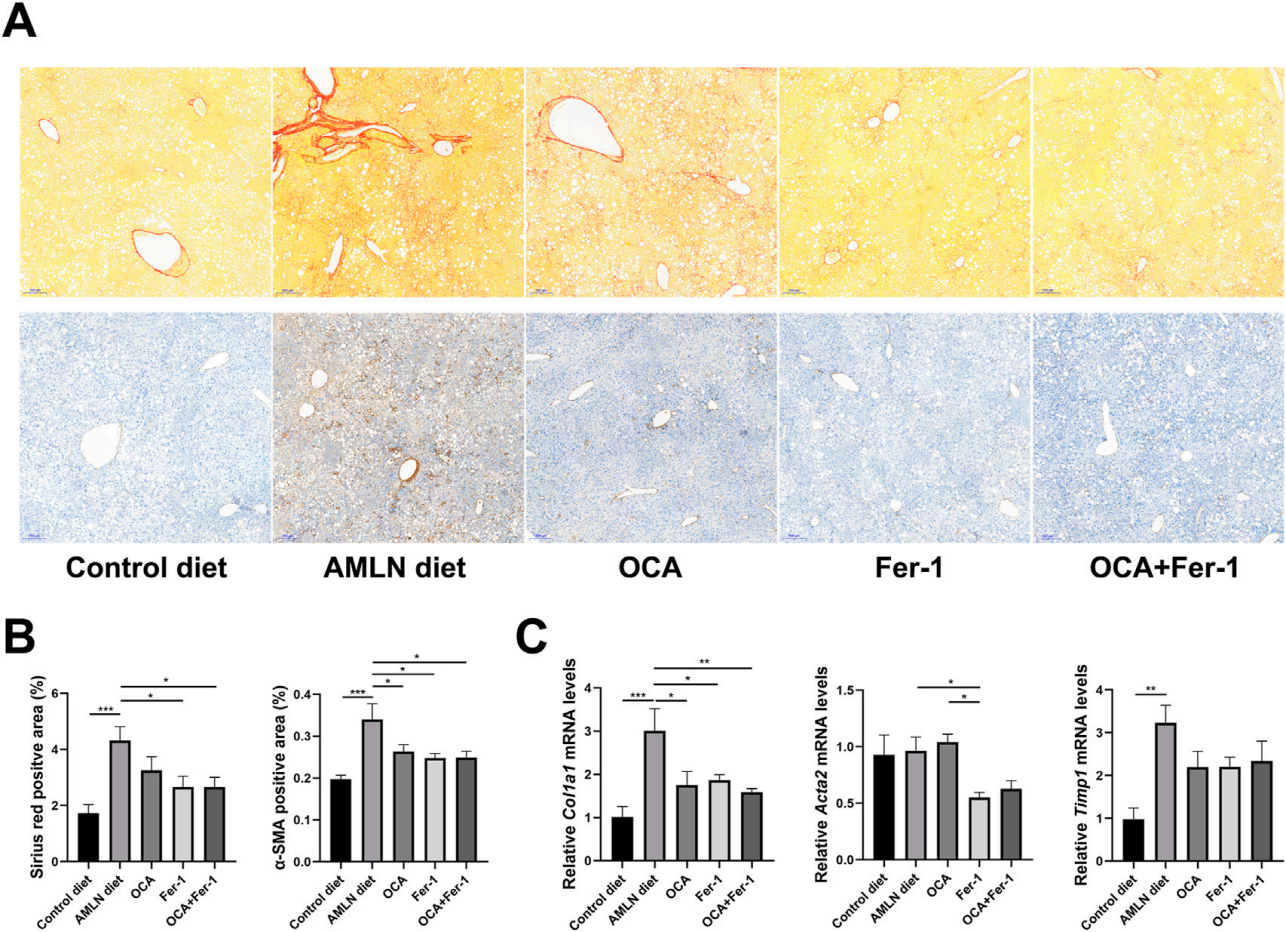
OCA and Fer-1 differentially inhibited activation of hepatic Kuppfer cell and HSCs **(A)** Representative sirius-red staining (upper) and α-SMA immunohistochemical (lower) staining of liver tissues (scale bar: 200 μm). **(B)** The positive area of Sirius red staining and α-SMA staining. **(C)** Relative expression of fibrosis related genes (*Col1a1*, *Acta2* and *Timp1*) in liver tissues. Data are shown as mean ± SEM. *p < 0.05, **p < 0.01, ***p < 0.001.

Sirius red staining and α-SMA immunohistochemical staining show that the mice in the AMLN diet group exhibit more severe fibrosis than those in the control diet group (Figure 3A). OCA and Fer-1 differentially decrease the degree of liver fibrosis and reduce the Sirius red positive area and α-SMA-positive area, but Fer-1 exhibits superior improvement (Figure 3B). The AMLN diet remarkably increases the relative expression of fibrosis-related genes (Col1a1 and Timp1) in the liver, while Fer-1 remarkably decreases the relative expression of Col1a1 and Acta2 (Figure 3C).

### 3.4 The combination of OCA and Fer-1 treatment protects mice against liver oxidative stress

**FIGURE 4:**
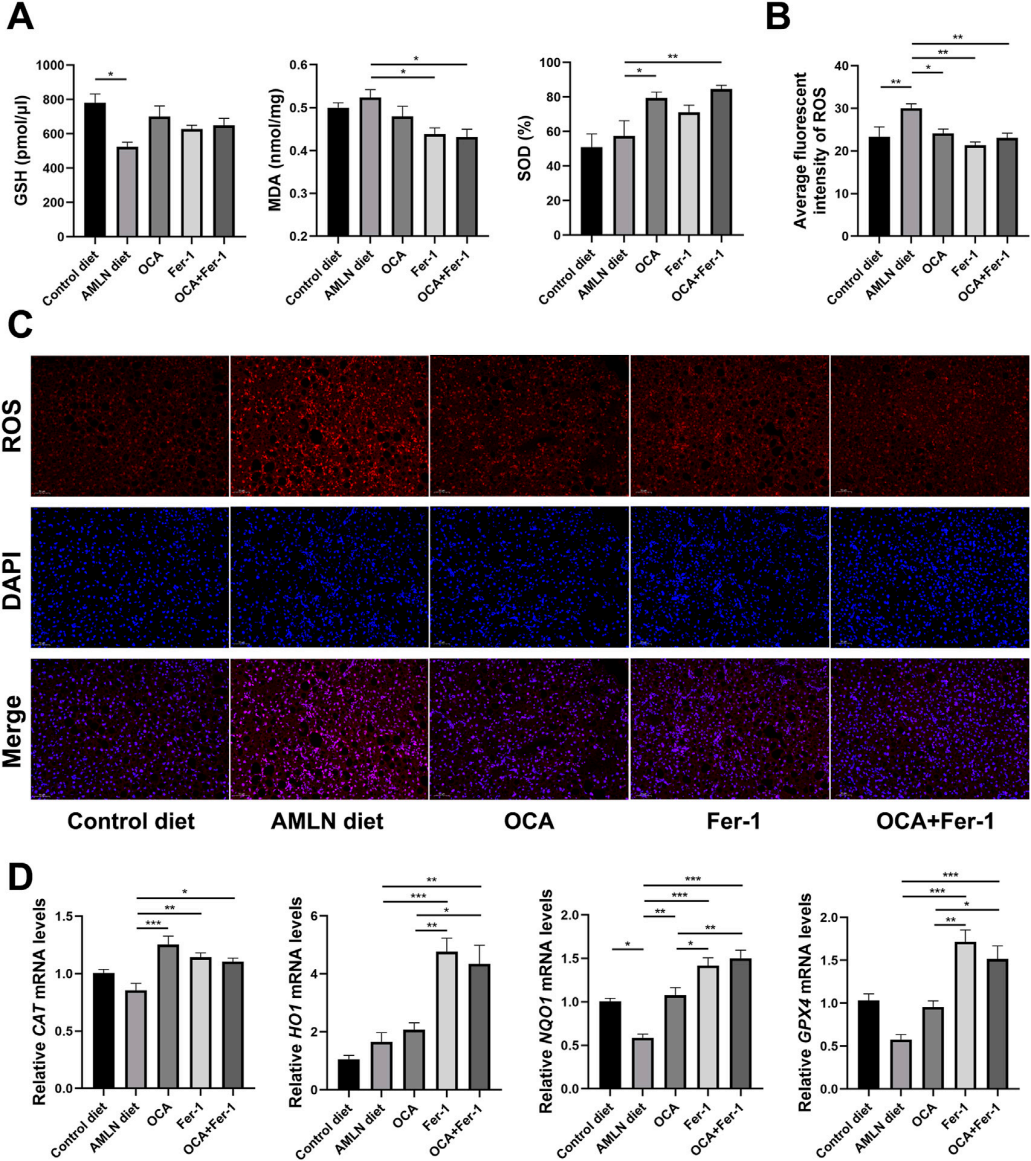
Combination of OCA and Fer-1 treatment protected mice against liver oxidative stress. **(A)** Levels of oxidative stress indicators (GSH, MDA and SOD) in the liver. **(B)** Average fluorescence intensity of ROS. **(C)** Dihydroethidium staining of liver tissues (scale bar: 50 μm). **(D)** Relative expression of oxidative stress related genes (*CAT*, *HO1*, *NQO1* and *GPX4*) in liver tissues. Data are shown as mean ± SEM. *p < 0.05, **p < 0.01, ***p < 0.001.

Liver oxidative stress assessment reveals that the AMLN diet significantly reduces the hepatic level of GSH, which could be partly reversed by the OCA and Fer-1 treatments. ELISA shows that OCA significantly increases the level of SOD, and Fer-1 remarkably decreases the level of MDA. Moreover, the combination of OCA and Fer-1 treatment markedly increases the SOD activity and decreases the level of MDA (Figure 4A). Consistent with this, the combination of OCA and Fer-1 treatment lowers the AMLN diet-induced hepatic ROS levels (Figure 4B, Figure 4C) and significantly elevates the expression of the anti-oxidant genes CAT, HO1, NQO1 and GPX4 in the liver (Figure 4D).

### 3.5 OCA and Fer-1 differentially reshape the intestinal microbiome

**FIGURE 5:**
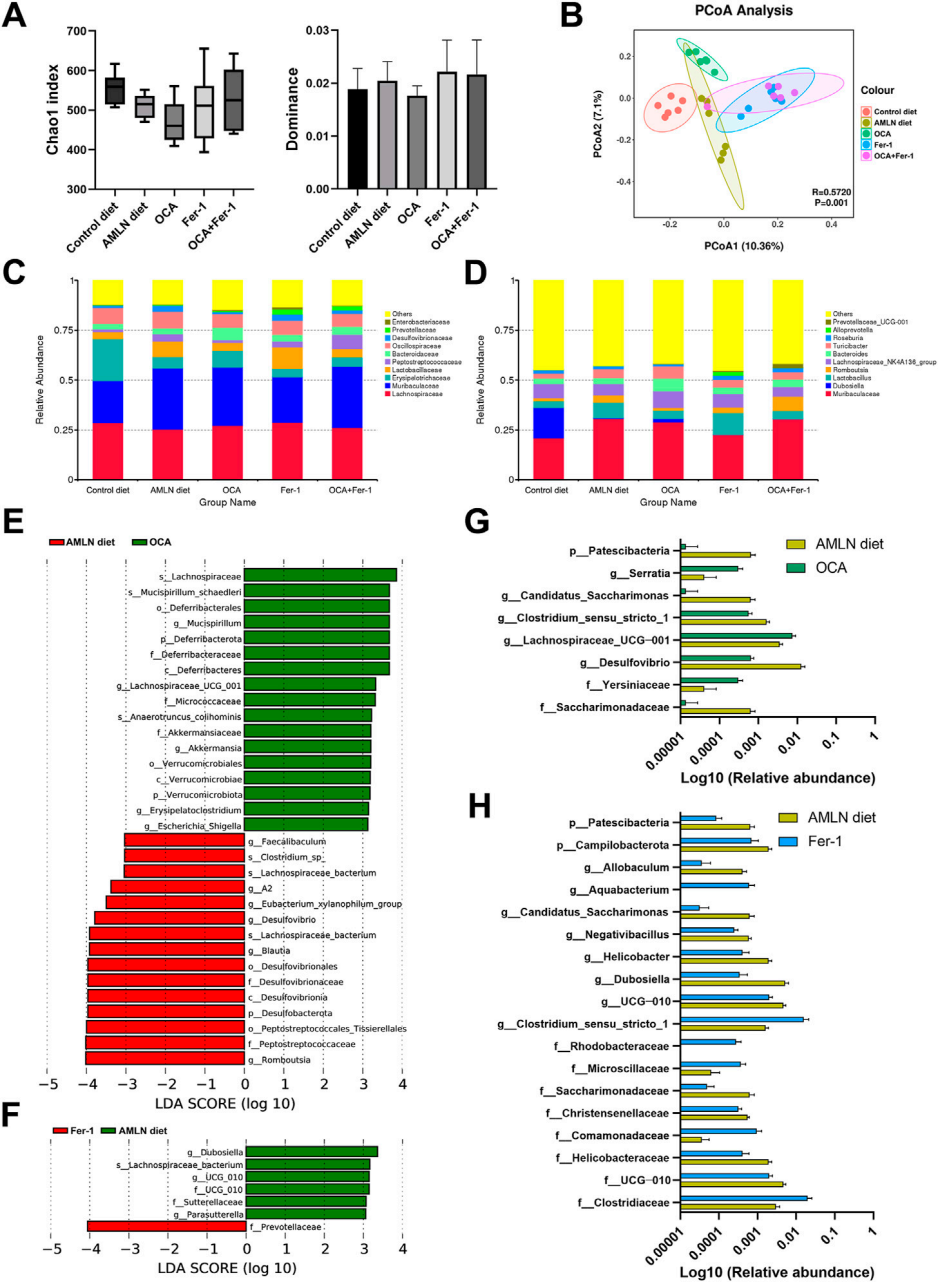
OCA and Fer-1 differentially reshaped intestinal microbiome. **(A)** alpha diversity indexes (Chao1 and dominance) of five groups. **(B)** Principal coordinates analysis (PCoA) based on weighted UniFrac distances. **(C)** Relative abundance of altered taxa at the family **(F)** and genus **(G)** levels. **(E–F)** LEfSe cladogram represents the taxa enriched in the groups. **(G)** Taxa with significant differences between the AMLN diet group with OCA group. **(H)** Taxa with significant differences between the AMLN diet group with Fer-1 group. Data are shown as mean ± SEM **(D)**.

**FIGURE 6:**
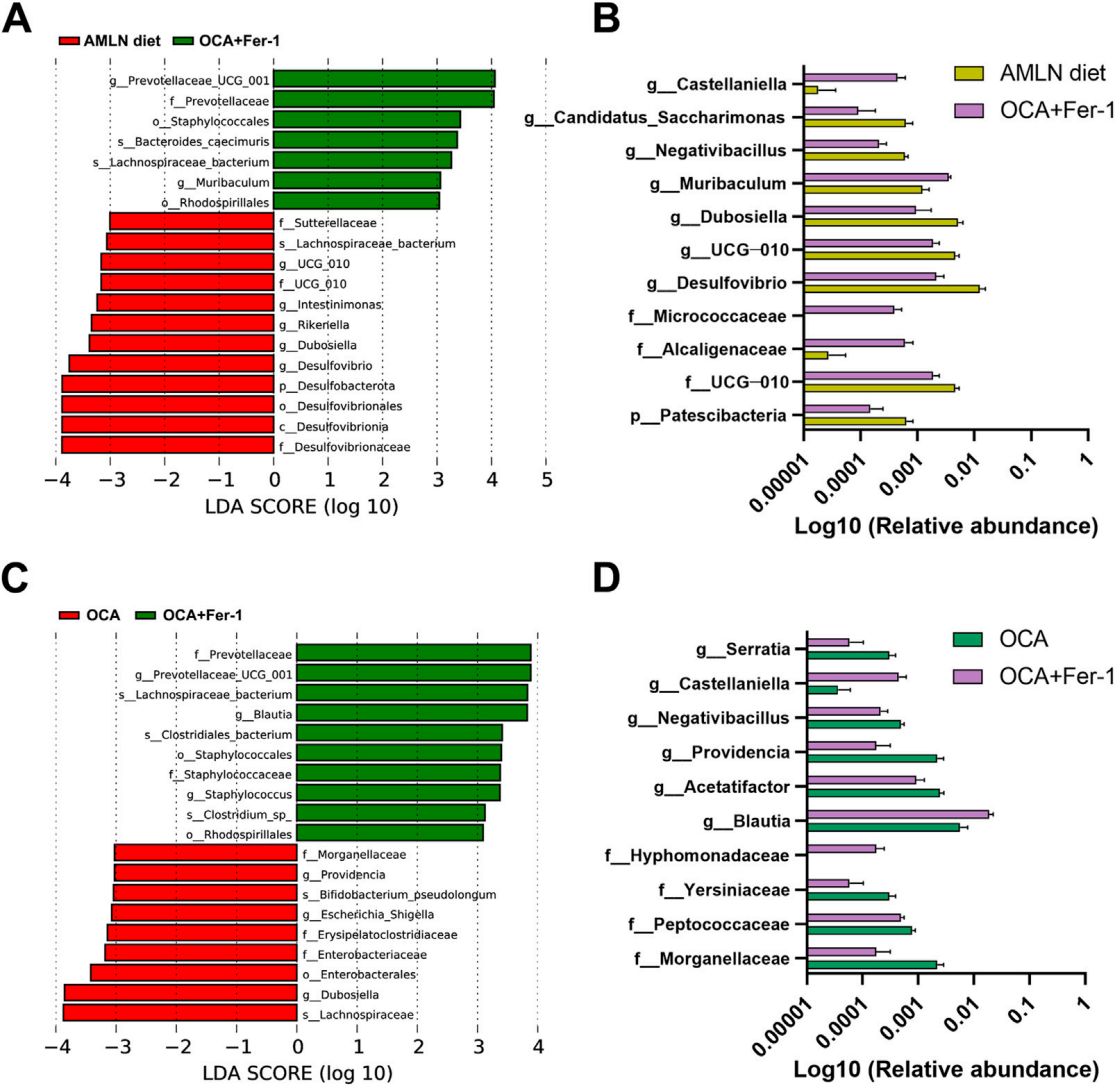
The combination of OCA and Fer-1 differentially reshaped intestinal microbiome. **(A)** LEfSe cladogram represents the taxa enriched in the AMLN diet group and the OCA + Fer-1 group. **(B)** Taxa with significant differences between the AMLN diet group and the OCA + Fer-1 group. **(C)** LEfSe cladogram represents the taxa enriched in the OCA group and the OCA + Fer-1 group. **(D)** Taxa with significant differences between the OCA group and the OCA + Fer-1 group. Data are shown as mean ± SEM.

There are downward trends in the Chao1 index and the dominance index in mice after OCA treatment compared to those in the AMLN diet group (Figure 5A). The PCoA analysis indicates obviously distinctive compositions of the microbial community among these five groups (Figures 5B–D). LEfSe analysis reveals that the AMLN diet increases the abundance of *Bacteroidota*, Muribaculaceae, *Desulfovibrionaceae*, *Peptostreptococcaceae*, *Romboutsia* and *Rikenellaceae* and decreases the abundance of *Erysipelotrichaceae*, *Dubosiella*, *Firmicutes*, *Bifidobacteriaceae*, *Actinobacteriota* and *NK4A214_group* (Supplementary Figure S4A). However, OCA treatment increases the abundance of *Mucispirillum*, *Deferribacteraceae*, *Lachnospiraceae_UCG_001*, *Micrococcaceae*, *Akkermansiaceae*, *Verrucomicrobiota* and *Escherichia_Shigella* and decreases the abundance of *Romboutsia*, *Peptostreptococcaceae*, *Desulfobacterota* and *Blautia* (Figure 5E); the taxa with significantly different abundances after OCA treatment are shown in Figure 5G. As shown in Figure 5F, the abundance of *Prevotellaceae* is increased after Fer-1 treatment, while the abundances of *Dubosiella*, *UCG_010*, *Sutterellaceae* and *Parasutterella* are decreased. Taxa with significantly different abundances after Fer-1 treatment are shown in Figure 5H. LEfSe analysis indicates that *Dubosiella*, *Rikenellaceae*, *Alistipes*, *Deferribacteraceae*, *Helicobacter* and *Akkermansia* are enriched in the OCA group, while *Lactobacillus*, *Prevotellaceae* and *Blautia* are enriched in the Fer-1 group (Supplementary Figure S4B). Mice that received the combination of OCA and Fer-1 shows increases in the abundances of *Prevotellaceae* and *Muribaculum* and decreases in the abundances of *Desulfovibrionaceae*, *Dubosiella*, *Rikenella*, *Intestinimonas*, and *UCG_010* compared to mice in the AMLN diet group (Figure 6A); however, compared to mice that received OCA treatment alone, mice that received the combination of OCA and Fer-1 shows increases in the abundances of *Prevotellaceae*, *Blautia*, *Staphylococcaceae*, and *Staphylococcus* and decreases in the abundances of *Dubosiella*, *Enterobacteriaceae*, *Erysipelatoclostridiaceae*, *Escherichia_Shigella*, *Providencia*, and *Morganellaceae* (Figure 6C). Taxa with significantly different abundances between the OCA + Fer-1 group and the AMLN diet group or the OCA + Fer-1 group and the OCA group are shown in Figures 6B,D, respectively.

### 3.6 OCA and Fer-1 differentially modulate the hepatic lipidome

**FIGURE 7:**
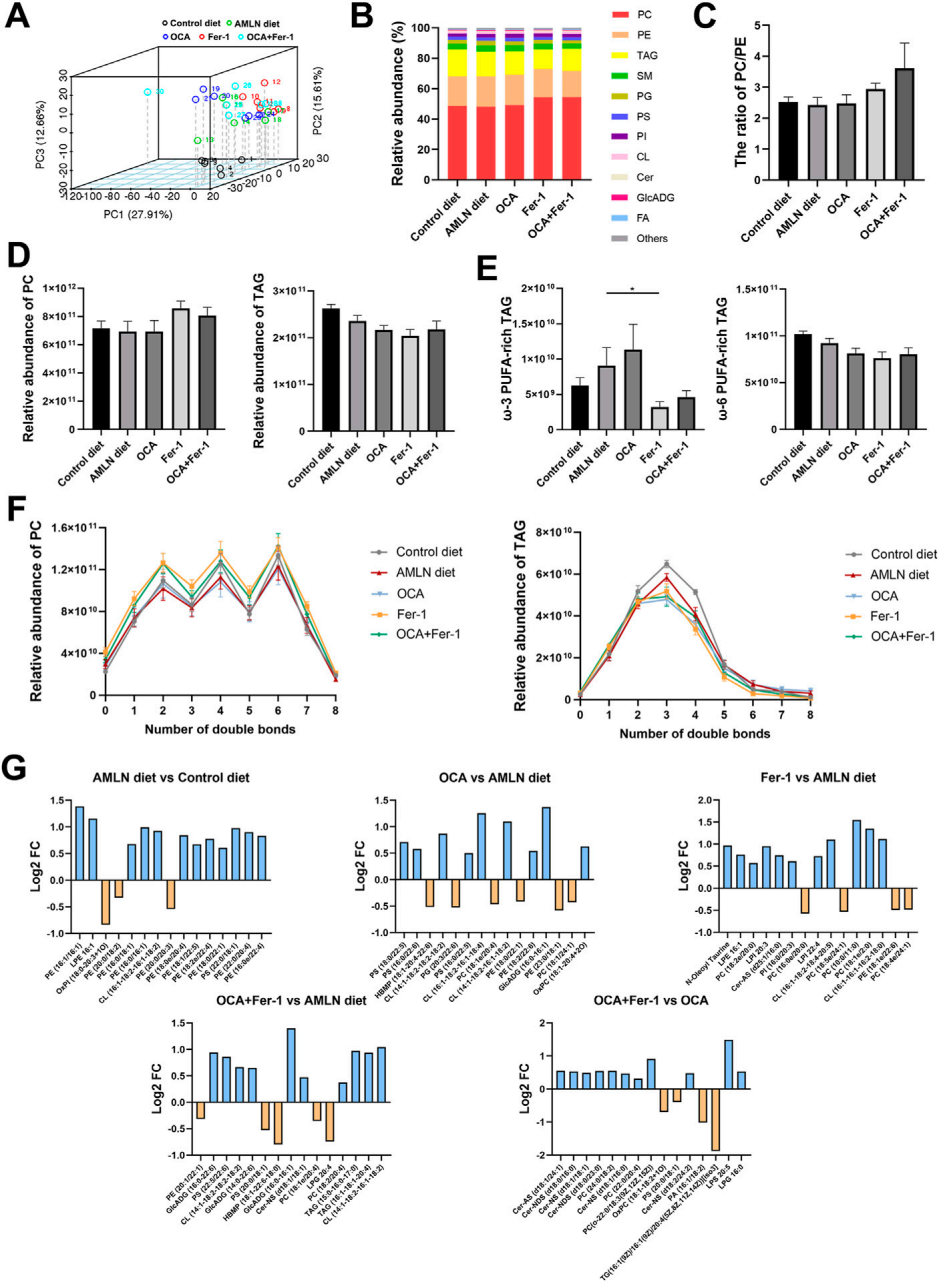
OCA and Fer-1 differentially modulated hepatic lipidome. **(A)** Principal Component Analysis between the five groups. **(B)** Relative abundance (%) of lipid subclass among five groups **(C)** The ratio of PC/PE. **(D)** Relative abundance of PC (left) and TAG (right). **(E)** Relative abundance of ω-3 PUFA -rich TAG (left) and ω-6 PUFA -rich TAG (right). **(F)** Relative abundance of PC (left) and TAG (right) with different amount of double bonds. **(G)** The top 15 most significant lipids between groups. Data are shown as mean ± SEM. *p < 0.05.

To assess lipotoxic levels in the NASH model, we conducted lipidomics analyses to identify specific changes in hepatic lipid profiles, and 1465 lipids are identified. PCA indicates apparent separation between the Control diet group and the AMLN diet group, as well as partial separation between these different treatment groups (Figure 7A). Lipid class composition shows that Fer-1 treatment increases the proportion of phosphatidylcholine (PC) and reduces the proportion of triacylglycerols (TAG) in liver tissues (Figures 7B,D). There is an upward trend of PC/phosphatidylethanolamine (PE) after Fer-1 treatment (the Fer-1 group and the OCA + Fer-1 group) (Figure 7C). Moreover, Fer-1 treatment decreases the relative abundance of ω-3 polyunsaturated fatty acid (PUFA)-rich TAG and ω-6 PUFA-rich TAG (Figure 7E). In addition, the increase in PC after Fer-1 treatment is more profound in lipids with a small amount of double bonds (Figure 7F), and the reduction in TAG with Fer-1 treatment is exclusively observed in lipids with 2-5 double bonds (Figure 7F). The top 15 most significantly different lipids among the groups are shown in Figure 7G, and the respective *p*-values are listed in the Supplementary Table S2.

## 4 Discussion

Currently, the primary approaches for treating NAFLD/NASH are based on lifestyle changes, including changes in diet and weight management, which require substantial self-control and coordination. Pharmacological support may benefit these patients who are unable to maintain a healthier lifestyle. Our study demonstrates the effects of OCA, Fer-1 and their combination on AMLN diet-induced NASH in mice as shown by changes in different pathological parameters. Notably, each of the three treatments has its own advantages.

Farnesoid X receptor (FXR), which is a receptor that is mainly expressed in the liver and gut, can regulate the synthesis, transport and reabsorption of bile acid, also takes a significant role in carbohydrate as well as lipid metabolism (Jiang et al., 2021). Thus, OCA, which activates FXR, is considered a promising treatment for NAFLD/NASH. In our research, we find that OCA administration markedly relieves liver lipid accumulation and lowers serum TC, TG and TBA levels in mice with AMLN diet-induced NASH. However, OCA exerts a mild effect on weight control and glucose homeostasis. In addition, OCA ameliorates the degree of hepatic fibrosis and reduces the gene expression of Col1a1. Type I collagen, which is one of the major extracellular matrix components, is closely associated with fibrogenesis (Tsukada et al., 2006). α-SMA, an activation marker of hepatic stellate cells, is overexpressed in NASH which was partly reversed by OCA, but the relative expression of *ACTA2* is not altered by OCA.

Ferroptosis has been proposed to be essential for the occurrence and progression of NAFLD/NASH, and this is verified in our experiment. In contrast to OCA, a ferroptosis inhibitor (Fer-1) markedly improves weight control, glucose homeostasis and insulin sensitivity. Notably, PYY, which is a gut-secreted hormone that exerts a strong impact on glucose and energy homeostasis (Mishra et al., 2016), is activated by Fer-1 treatment, and *PPAR*γ mRNA expression is also elevated after Fer-1, which could regulate glucose and lipid homeostasis (Janani and Ranjitha Kumari, 2015). Moreover, Fer-1 exerts a better effect on reducing transaminase levels than OCA, which indicates that Fer-1 exhibits a superior impact on protecting hepatocytes from lipotoxicity. However, Fer-1 is not as effective as OCA in reducing blood lipid levels and alleviating liver lipid accumulation. Regarding the antifibrotic effect, Fer-1 exhibits a notable impact in that it not only reduces the gene expression of *Col1a1* but also partially inhibits the activation of Kupffer cells and HSCs.

For the combination of OCA and Fer-1, we notice that it exerts excellent effects in anti-inflammation and antioxidation. Researchers found that OCA ameliorated NASH by directly inhibiting NLRP3 inflammasome activation in macrophages, which could reduce lipid accumulation (Huang et al., 2021). Previous work has demonstrated the anti-inflammatory effect of Fer-1 on NASH (Liu et al., 2021a). Moreover, OCA increases antioxidation capacity; in particular, it elevates the levels of SOD and CAT. Consistently, researchers have confirmed the antioxidative effects of OCA as a result of FXR activation, and OCA pretreatment markedly attenuated valproic acid-induced hepatic lipid peroxidation and endoplasmic reticulum stress in mice (Gai et al., 2020). Moreover, Fer-1 treatment increases the levels of the GSH-dependent enzyme glutathione peroxidase-4 (*GPX4*), which could reduce lipid peroxides to alcohols (Shojaie et al., 2020), and increases the antioxidation capacity, especially by elevating the levels of *CAT*, *NQ O 1* and *H O 1*. *NQ O 1* and *H O 1* are downstream genes of the antioxidant gene *Nrf2* that regulates lipid metabolism, protects mice against lipotoxicity and controls fibrogenesis and carcinogenesis during steatohepatitis (Yan et al., 2018; Lee et al., 2020; Mohs et al., 2021). Therefore, OCA and Fer-1 exerts anti-inflammation and antioxidation effects through different pathways. When OCA and Fer-1 are combined, their respective effects are exerted, which may explain why the combination of OCA and Fer-1 exerts the best anti-inflammatory and antioxidative stress effects.

Several studies have indicated that the intestinal microbiota participates in NAFLD/NASH (Leung et al., 2016; Kolodziejczyk et al., 2019). Wang B et al. found that *Bacteroidetes* was enriched while *Firmicutes* was depleted in NAFLD patients (Wang et al., 2016). Consistently, an increase in *Bacteroidota* and a decrease in *Firmicutes* are observed after AMLN diet feeding; moreover, dysbiosis occurs, and the abundances of beneficial bacteria, such as *Bifidobacterium* and *Dubosiella,* are decreased. On the one hand, researchers discovered that the depletion of *Firmicutes* included SCFA-producing *Lachnospiraceae* (Wang et al., 2016), and this effect could be reversed by OCA treatment in our experiment. Moreover, the abundances of beneficial bacteria, such as *Akkermansia* and *Mucispirillum,* are increased after OCA treatment. Notably, *Akkermansia* is reported to decrease serum triglyceride levels and prevent NAFLD in animals (Kim et al., 2020; Juárez-Fernández et al., 2021). On the other hand, Fer-1 treatment increases the abundance of *Lactobacillales*, which could improve NAFLD by modulating the gut metagenomic and metabolic environment, especially the tryptophan pathway (Yu et al., 2021). The abundance of the new functional genus *Blautia* is also increased by Fer-1, which was proven to be positively correlated with glucose and lipid homeostasis improvements (Liu et al., 2021b).

Previous human studies demonstrated significant increases in the total levels of hepatic saturated fatty acids (SFAs) and polyunsaturated fatty acids (PUFAs) in NAFLD/NASH patients, whereas the levels of *γ*-linolenic acid (C18:3n-6) and arachidonic acid (C20:4n-6) were significantly decreased (Puri et al., 2007). PC and PE are major phospholipids in mammalian membranes, and researchers have found that the ratio of PC/PE takes an important role in the progression of steatohepatitis and that it is lower in NASH patients (Li et al., 2006). Therefore, the combination of OCA and Fer-1 could increase the ratio of PC/PE and partially reverse the progression of steatohepatitis. It is widely believed that excessive TAG accumulation is a basic characteristic of NAFLD (Donnelly et al., 2005), and this accumulation could be decreased by Fer-1 treatment. The ratio of *ω*-6 to *ω*-3 PUFAs is recently shown to be elevated due to the increased dietary intake of *ω*-6 PUFA, and it is considered to promote the development of NAFLD/NASH (Araya et al., 19792004; Masoodi et al., 2021). However, the Fer-1-mediated decrease in TAG also includes decreases in TAGs containing *ω*-3 PUFAs eicosapentanoic (20:5n-3) and docosahexanoic (22:6n-3). In contrast, there is a trend of increased *ω*-3 PUFA-rich TAG after OCA treatment, and these molecules have been shown to have anti-inflammatory and metabolic properties (Calder, 2003; Mori et al., 2003).

There are some limitations in our study. Firstly, our research about effects of OCA, Fer-1 and their combination on NASH is not thorough enough, the mechanisms under these effects are still not clear. For example, the key lipid or microbiota that plays a crucial role in the disease which may influence the progression of NASH is not found yet. Secondly, the Fer-1 is a new promising drug according to various animal researches on different diseases but few studies have revealed its safety on animal and human. Therefore, the safety of Fer-1 as a treatment remains a problem worth noting. Thirdly, our study is based on mouse model, nevertheless the effect of Fer-1 on NASH patients requires clinical trials, that is, a large number of researches about the efficacy of Fer-1 in human are needed in the future.

In conclusion, our study compares the efficacy of two promising drugs, OCA and Fer-1, for the treatment of NAFLD/NASH based on assessing various parameters, including metabolic status, inflammation, fibrosis, oxidative stress, the microbiota and the lipidome. OCA has a remarkable effect on improving liver lipid accumulation and liver fibrosis, enriching beneficial bacteria, such as *Lachnospiraceae*, *Akkermansia* and *Mucispirillum*, and increasing the levels of *ω*-3 PUFA-rich TAGs. Fer-1 plays a crucial role in weight control, glucose homeostasis and insulin sensitivity, as well as alleviating liver fibrosis and lowering hepatic TAG. The combination of OCA and Fer-1 exerts excellent anti-inflammatory and antioxidative stress effects and increases the abundance of the new functional genus *Blautia* as well as the ratio of PC/PE. Different drug combinations exert their own effects, and each has its advantages. Our research provides reference about the effects of OCA, Fer-1 and their combination on NAFLD/NASH, thus making it a possible strategy to treat NAFLD/NASH in the future.

## Data Availability

The original contributions presented in the study are included in the article/Supplementary Material, further inquiries can be directed to the corresponding author.
