# Supplementary material for: Obeticholic acid and ferrostatin-1 differentially ameliorate non-alcoholic steatohepatitis in AMLN diet-fed ob/ob mice
Source: Front Pharmacol. 2022 Dec 16;13:1081553. doi: 10.3389/fphar.2022.1081553 (PMC9800415; doi:10.3389/fphar.2022.1081553)
Supplement: Supplementary file 1 [file DataSheet1.docx]

**Supplemental files**

**Supplemental Methods:**

**16S rRNA Sequencing of Cecal Contents**

Total genomic DNA was extracted from cecal contents using the CTAB/SDS method and diluted to 1 ng/μL with sterile water, and amplicon generation was conducted with primers (V4: 515F-806R) and barcodes. After DNA detection, a Qiagen Gel Extraction Kit (Qiagen, Dusseldorf, Germany) was used to purify the PCR products. Then, an NEBNext® Ultra™ IIDNA Library Prep Kit (Cat No. E7645, USA) was used to generate the sequencing libraries following the manufacturer’s protocols. ASV denoising, species annotation and multiple sequence alignment were performed using QIIME2 software. The alpha diversity (the Chao1 index and the dominance index, etc.) and the beta diversity (principal coordinate analysis (PCoA), etc.) were calculated in QIIME2 software. R software (Version 3.5.3) was used to perform MetaStat and T test analyses to identify the significantly different species at each taxonomic level (phylum, class, order, family, genus, species). To identify biomarkers, LEfSe software (Version 1.0) was used to perform LEfSe analysis (LDA score threshold: 3). The 16S sequencing data were available on the Sequence Read Archive (SRA) database (BioProject number PRJNA 884173).

**Lipidomics Analysis of Liver Tissues**

Liver tissue powder (100 mg) was resuspended in 0.75 mL methanol by vortexing and incubated for 1 h at room temperature in a shaker after adding 2.5 mL of MTBE. MS-grade water (0.625 mL) was added to induce phase separation, and the upper phase was collected after 10 min of incubation and centrifugation. Organic phases were dried and dissolved in 100 μL of isopropanol for storage, and then a Vanquish UHPLC system (Thermo Fisher, Massachusetts, USA) coupled with an Orbitrap Q Exactive^TM^ HF mass spectrometer (Thermo Fisher, Massachusetts, USA) was used to perform UHPLC‒MS/MS analyses. The generated raw data files were processed to perform peak alignment, peak picking and quantitation for each metabolite by Compound Discoverer 3.1 (Thermo Fisher, Massachusetts, USA). Then, peak intensities were normalized to the total spectral intensity and matched with the Lipidmaps and Lipidblast database to obtain accurate qualitative and relative quantitative results. Principal components analysis (PCA) was performed at metaX, univariate analysis (t test) was used to calculate the statistical significance (P value), and the metabolites characterized by VIP > 1, P value< 0.05, and fold change≥ 2 or FC≤ 0.5 were considered to be differential metabolites.

**Table S1** Primer pairs for Real-time Quantitative PCR analysis of indicated genes. *GADPH* was used as internal control.

| **Gene** | **Forward sequence (5’-3’)** |  | **Reserve sequence (3’-5’)** |
| --- | --- | --- | --- |
| *GAPDH* | AGGTCGGTGTGAACGGATTTG |  | TGTAGACCATGTAGTTGAGGTCA |
| *PPAR-γ* | TTGCTGAACGTGAAGCCCATCGAGG |  | GTCCTTGTAGATCTCCTGGAGCAG |
| *Col1a1* | CACCCCAATCTGGTTCCCTC |  | CATAAGCCAAGTGGGCAGGA |
| *Acta2* | GGCACCACTGAACCCTAAGG |  | ACAATACCAGTTGTACGTCCAGA |
| *Timp1* | CAGATACCATGATGGCCCCC |  | CGCTGGTATAAGGTGGTCTCG |
| *CAT* | GAAGGACCGTGTTTGGTTGC |  | CCGCTGGCGCTTTTATTGTT |
| *GPX4* | CCTGGTCTGGCAGGCAC |  | GCTAGAGATAGCACGGCAGG |
| *NQO1* | AGCCAATCAGCGTTCGGTAT |  | GCCTCCTTCATGGCGTAGTT |
| *HO1* | GGGAACATTTTTGGGGCGAC |  | TCCTCGATGTTGGCCCTTTC |

**Table S2** The top 15 most significantly different lipids among the groups and their respective *p*-values.

| **AMLN diet vs Control diet** | | **OCA vs AMLN diet** | | **Fer-1 vs AMLN diet** | |
| --- | --- | --- | --- | --- | --- |
| **Name** | **Pvalue** | **Name** | **Pvalue** | **Name** | **Pvalue** |
| PE (16:1/16:1) | 2.30E-05 | PS (18:0/22:5) | 0.000123 | N-Oleoyl Taurine | 9.52E-05 |
| LPE 16:1 | 2.56E-05 | PS (16:0/22:6) | 0.000142 | LPE 16:1 | 0.000221 |
| OxPI (18:0-20:3+1O) | 2.58E-05 | HBMP (18:1-20:4-22:6) | 0.00052 | PC (18:2e/20:0) | 0.001341 |
| PE (20:0/18:2) | 3.40E-05 | CL (14:1-18:2-18:2-18:2) | 0.000579 | LPI 20:3 | 0.001889 |
| PE (16:0/18:1) | 5.68E-05 | PG (20:3/22:6) | 0.000793 | Cer-AS (d25:1/16:0) | 0.002003 |
| PE (16:0/16:1) | 5.82E-05 | PS (16:0/22:5) | 0.000819 | PI (16:0/20:3) | 0.002486 |
| CL (16:1-18:2-16:1-18:2) | 8.41E-05 | CL (16:1-18:2-16:1-18:4) | 0.001227 | PC (16:0e/20:0) | 0.002602 |
| PE (20:0/20:3) | 0.000107 | PC (18:1e/20:4) | 0.001253 | LPI 22:4 | 0.0027 |
| PE (18:0e/20:4) | 0.000144 | CL (14:1-18:2-16:1-18:2) | 0.001279 | CL (16:1-18:2-18:4-20:5) | 0.002861 |
| PE (18:1/22:5) | 0.000161 | PE (18:0/22:1) | 0.001307 | PC (18:5e/24:1) | 0.00291 |
| PE (18:2e/22:4) | 0.000164 | PE (18:2/22:6) | 0.001469 | PC (10:0/11:0) | 0.003213 |
| PE (18:0/22:1) | 0.000167 | GlcADG (16:0-16:1) | 0.001508 | PC (18:1e/2:0) | 0.003618 |
| PS (22:0/18:1) | 0.0002 | PE (23:0/18:1) | 0.001538 | CL (16:1-16:1-16:2-18:0) | 0.003765 |
| PE (22:0/20:4) | 0.000251 | PC (18:1/24:1) | 0.001842 | PE (18:1e/22:6) | 0.004186 |
| PE (16:0e/22:4) | 0.000265 | OxPC (18:1-20:4+2O) | 0.003381 | PC (18:4e/24:1) | 0.004432 |

| **OCA+Fer-1 vs AMLN diet** | | **OCA+Fer-1 vs OCA** | |
| --- | --- | --- | --- |
| **Name** | **Pvalue** | **Name** | **Pvalue** |
| PE (20:1/22:1) | 0.000828 | Cer-AS (d18:1/24:1) | 0.000693 |
| GlcADG (16:0-22:6) | 0.001013 | Cer-NDS (d18:0/16:0) | 0.00129 |
| PS (22:5/22:6) | 0.002041 | Cer-NS (d18:1/18:1) | 0.001347 |
| CL (14:1-18:2-18:2-18:2) | 0.002529 | Cer-NDS (d18:0/23:0) | 0.002578 |
| GlcADG (14:0-22:6) | 0.003524 | PC (24:0/18:2) | 0.002893 |
| PS (20:0/18:1) | 0.004646 | Cer-NS (d18:1/16:0) | 0.003314 |
| HBMP (18:1-22:6-18:0) | 0.004867 | PC (22:0/20:4) | 0.011648 |
| GlcADG (16:0-16:1) | 0.00548 | PC(o-22:0/18:3(9Z,12Z,15Z)) | 0.01453 |
| Cer-NS (d18:1/18:1) | 0.00564 | OxPC (18:1-18:2+1O) | 0.017374 |
| PC (18:1e/20:4) | 0.006133 | PS (20:0/18:1) | 0.01843 |
| LPG 20:4 | 0.006583 | Cer-NS (d18:2/24:2) | 0.020598 |
| PC (18:2/20:4) | 0.007791 | PA (16:1/18:2) | 0.027788 |
| TAG (15:0-16:0-17:0) | 0.007956 | TG(16:1(9Z)/16:1(9Z)/20:4(5Z,8Z,11Z,14Z))[iso3] | 0.028135 |
| TAG (16:1-18:1-20:4) | 0.00833 | LPS 20:5 | 0.029183 |
| CL (14:1-18:2-16:1-18:2) | 0.008817 | LPG 16:0 | 0.031154 |

**Supplemental Figures:**

**
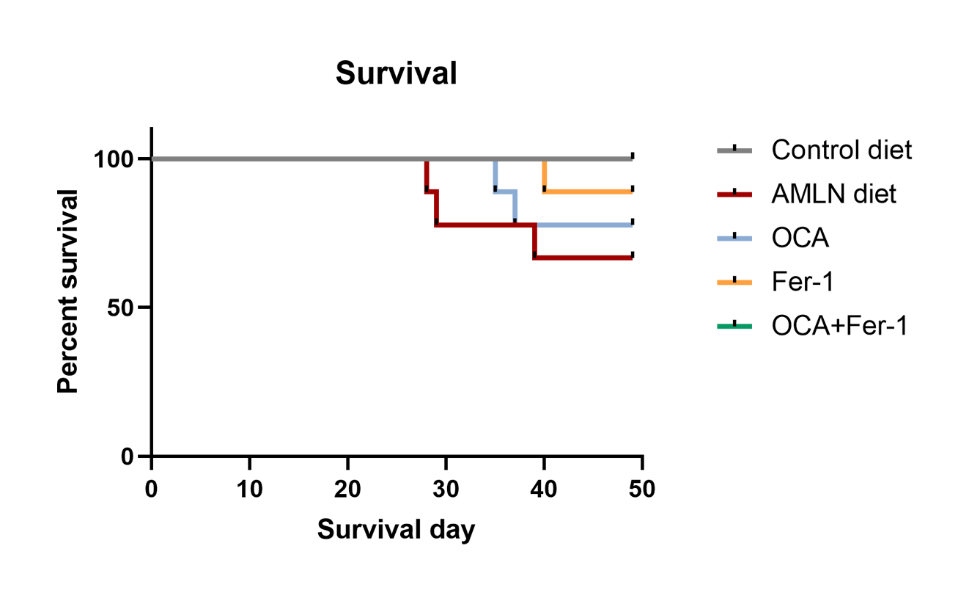
**

Figure S1. Survival curve of mice in five groups (Survival days are counted from treatments were firstly given).

**
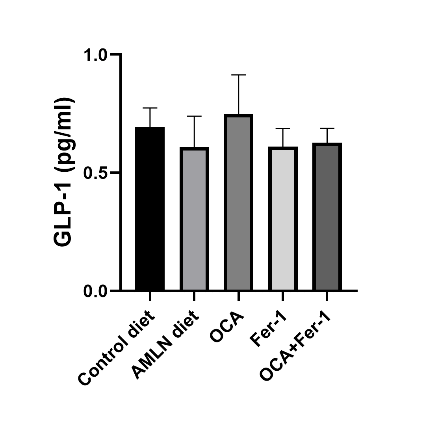
**

Figure S2. Levels of GLP-1 in the liver. Data are shown as mean ± SEM.


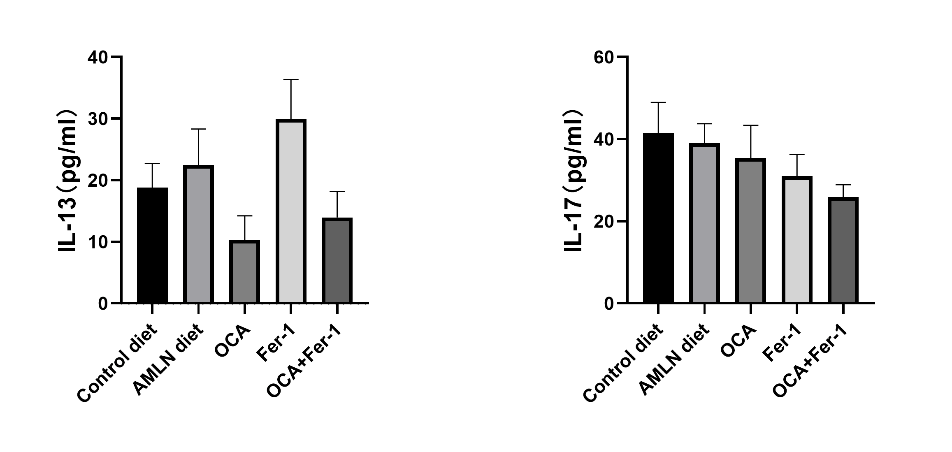


Figure S3. Serum cytokine levels of IL-13 (left) and IL-17 (right). Data are shown as mean ± SEM.


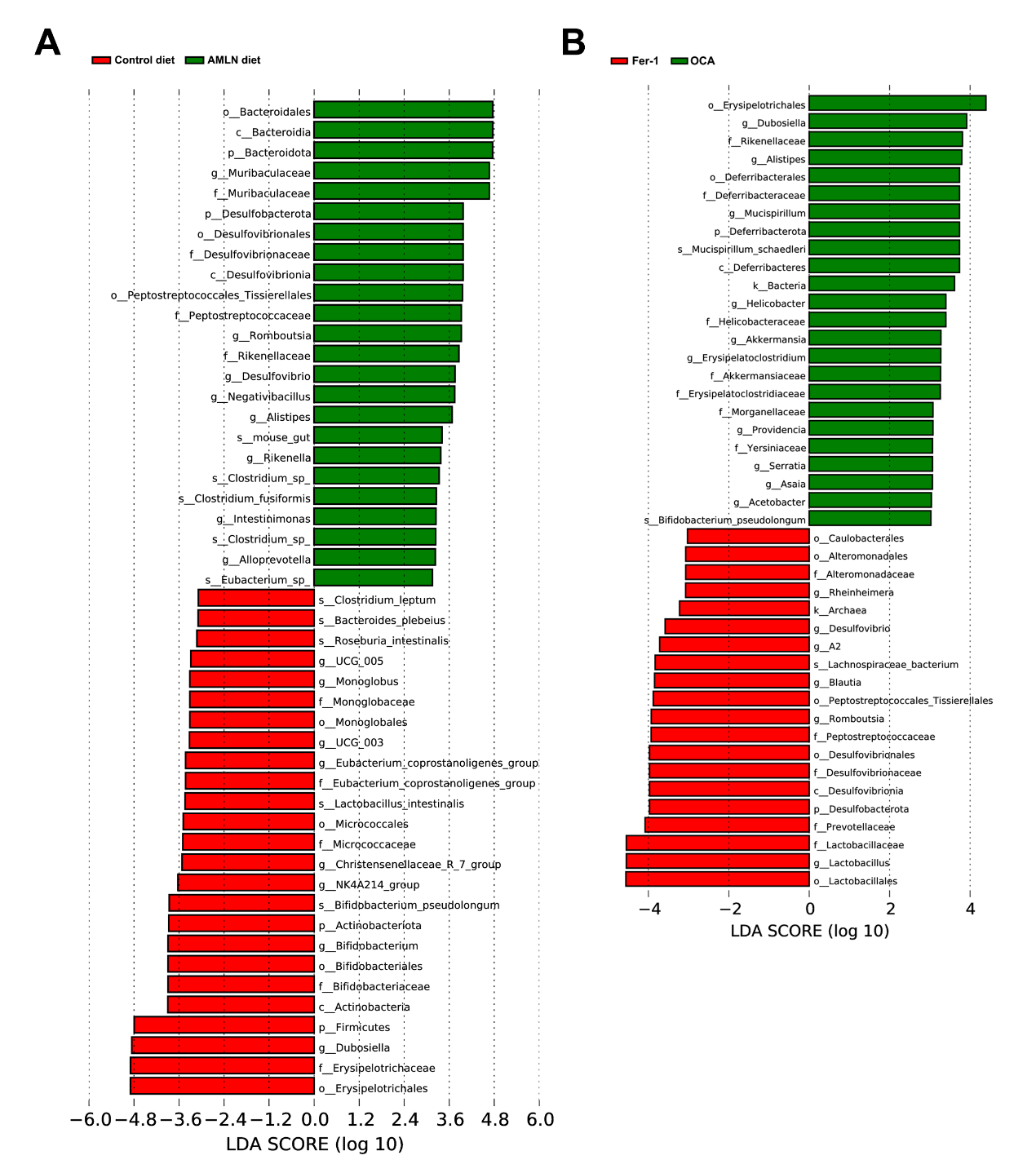


Figure S4. The treatment of OCA and Fer-1 differentially reshaped intestinal microbiome. (A) LEfSe cladogram represents the taxa enriched in the Control diet group and the AMLN diet group. (B) LEfSe cladogram represents the taxa enriched in the Fer-1 group and the OCA group. Data are shown as mean ± SEM.
